# Viperin inhibits rabies virus replication via reduced cholesterol and sphingomyelin and is regulated upstream by TLR4

**DOI:** 10.1038/srep30529

**Published:** 2016-07-26

**Authors:** Hai-Bo Tang, Zhuan-Ling Lu, Xian-Kai Wei, Tao-Zhen Zhong, Yi-Zhi Zhong, Ling-Xuan Ouyang, Yang Luo, Xing-Wei Xing, Fang Liao, Ke-Ke Peng, Chao-Qian Deng, Nobuyuki Minamoto, Ting Rong Luo

**Affiliations:** 1 State Key Laboratory for Conservation and Utilization of Subtropical Agro-bioresources, Guangxi University, Nanning 530004, Guangxi, China; 2 Laboratory of Animal Infectious Diseases, College of Animal Sciences and Veterinary Medicine, Guangxi University, Nanning 530004, Guangxi, China

## Abstract

Viperin (virus inhibitory protein, endoplasmic reticulum-associated, IFN-inducible) is an interferon-inducible protein that mediates antiviral activity. Generally, rabies virus (RABV) multiplies extremely well in susceptible cells, leading to high virus titres. In this study, we found that viperin was significantly up-regulated in macrophage RAW264.7 cells but not in NA, BHK-21 or BSR cells. Transient viperin overexpression in BSR cells and stable expression in BHK-21 cells could inhibit RABV replication, including both attenuated and street RABV. Furthermore, the inhibitory function of viperin was related to reduce cholesterol/sphingomyelin on the membranes of RAW264.7 cells. We explored the up-stream regulation pathway of viperin in macrophage RAW264.7 cells in the context of RABV infection. An experiment confirmed that a specific Toll-like receptor 4 (TLR4) inhibitor, TAK-242, could inhibit viperin expression in RABV-infected RAW264.7 cells. These results support a regulatory role for TLR4. Geldanamycin, a specific inhibitor of interferon regulatory factor 3 (IRF3) (by inhibiting heat-shock protein 90 (Hsp90) of the IRF3 phosphorylation chaperone), significantly delayed and reduced viperin expression, indicating that IRF3 is involved in viperin induction in RAW264.7 cells. Taken together, our data support the therapeutic potential for viperin to inhibit RABV replication, which appears to involve upstream regulation by TLR4.

Rabies is a worldwide zoonotic disease that causes a fatal infection of the central nervous system. Globally, it is responsible for more than 70,000 human deaths annually (http://www.oie.int/animal-health-in-the-world/rabies-portal/). To date, rabies is still an incurable disease with a mortality rate of almost 100%. The estimated annual cost for treatment or therapy of post-exposure to rabies by either dog or cat bites is approximately $12.4 billion^1^. Rabies continues to threaten global public health.

The innate immune system is an evolutionarily conserved system of defence against microbial infections^2^. One of the key cytokines released by host cells in response to the presence of pathogens such as viruses, bacteria, parasites or tumour cells is interferon (IFN). Type I IFN (IFN-α/β) is essential for immune defence against viruses and binds to the type I IFN receptor to induce the expression of hundreds of interferon-stimulated genes (ISGs). There are reports that RABV infection activates interferon gene expression in the brain^3^,^4^. Many ISGs limit viral replication. Viperin (virus inhibitory protein, endoplasmic reticulum-associated, IFN-inducible) is a type of ISG and is highly conserved from lower vertebrates to mammals. It has direct antiviral activity and plays an emerging role in modulating innate immune signalling^5^. Viperin is strongly induced in a variety of cells by type I/II IFNs and a broad range of viruses, poly(I:C), dsRNA, viral DNA, and lipopolysaccharides (LPS)^6^,^7^,^8^,^9^,^10^. Additionally, vesicular stomatitis virus (VSV)^11^, hepatitis C virus (HCV)^12^, and influenza A virus^13^ induce viperin expression in various cell lines. Viperin regulation by both IFN-dependent and IFN-independent pathways has been reported^8^,^9^,^11^. However, the antiviral mechanism of viperin is still unknown. Viruses seem to induce viperin expression either directly or through IFN induction. It is likely that viruses and IFN induce viperin through different mechanisms.

Therefore, we explored the capacity of viperin to function as an antiviral molecule against RABV and the mechanistic interaction between RABV and viperin in RAW264.7 cells. Viperin could inhibit both attenuated and street RABV replication and release by hindering viral budding and disrupting cholesterol/sphingomyelin in the RAW264.7 cell membrane. Additionally, the upstream regulation of viperin is regulated by Toll-like receptor (TLR) 4. These findings not only furthered our functional understanding of viperin but also provided evidence in support of this molecule as a new therapeutic target against rabies.

## Results

### Viperin is highly induced in RABV-infected macrophage RAW264.7 cells

**Figure 1:**
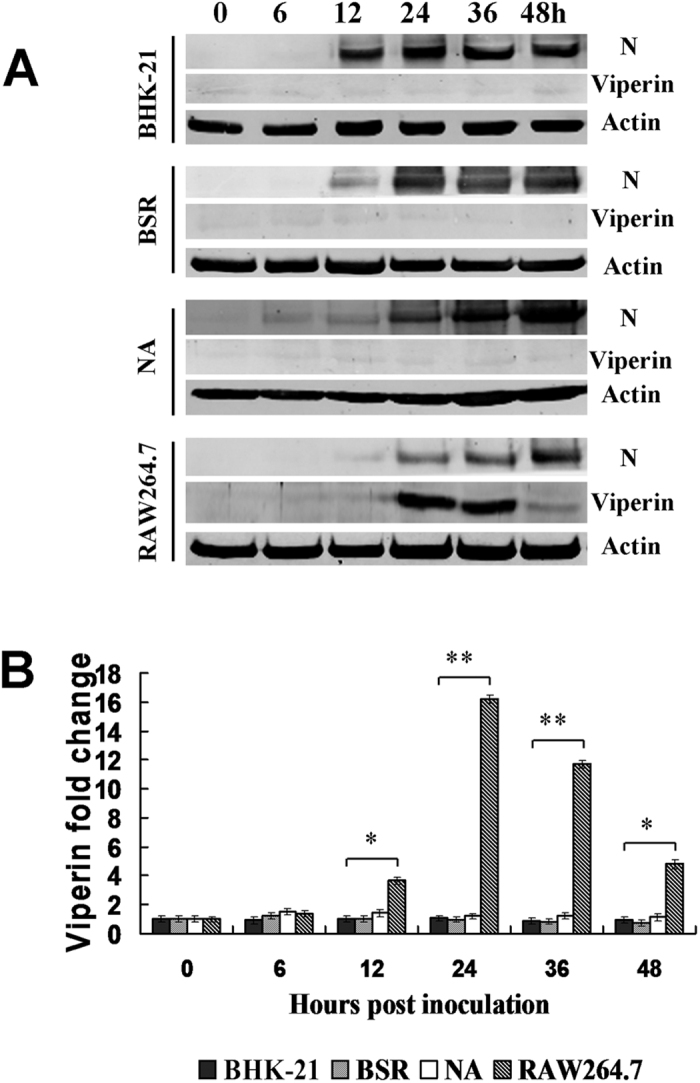
Viperin is induced in macrophage RAW264.7 cells during RABV infection. (**A**) Viperin levels as detected by Western blot in BHK-21, BSR, NA and RAW264.7 cell lines infected with rRC-HL at an MOI of 0.1 over time. RABV nucleoprotein (N) is defined as “N”. (**B**) Viperin/actin ratios over time in cell lines after rRC-HL infection.

Viperin is highly induced in RABV-infected, TLR3-positive human neurons^4^. Viperin can be categorized as an antiviral protein^14^,^15^,^16^. We hypothesized that viperin might preferentially inhibit RABV replication in RAW264.7 cells. To evaluate this possibility, Western blot analyses were performed to detect viperin expression upon RABV infection in cell lines. Fortunately, we unexpectedly found that high levels of viperin were induced in RAW264.7 cells infected with attenuated rRC-HL at 24 hours post-inoculation (hpi), 16-fold higher than that in NA, BHK-21 and BSR cells, in which viperin was either weakly detected or not expressed at all (Fig. 1A,B).

### Inhibition of RABV replication in transiently viperin-expressing BSR cells and stably viperin-expressing BHK-21 cells

To determine whether viperin inhibits RABV replication, BSR cells that transiently expressed viperin were exposed to RABV 12 h after transfection. Viral titres in the viperin-expressing BSR cells (4.68 × 10^4^ fluorescent focus units [FFU]/mL, 1.32 × 10^6^ FFU/mL and 9.77 × 10^6^ FFU/mL) were significantly lower than titres from transfected empty vector pcDNA3.0 BSR cells (3.02 × 10^5^ FFU/mL, 3.31 × 10^6^ FFU/mL and 2.00 × 10^7^ FFU/mL) at 24, 36, and 48 hpi, respectively. In addition, RABV N, P and M protein expression levels were significantly reduced following viperin expression (data not shown).

**Figure 2:**
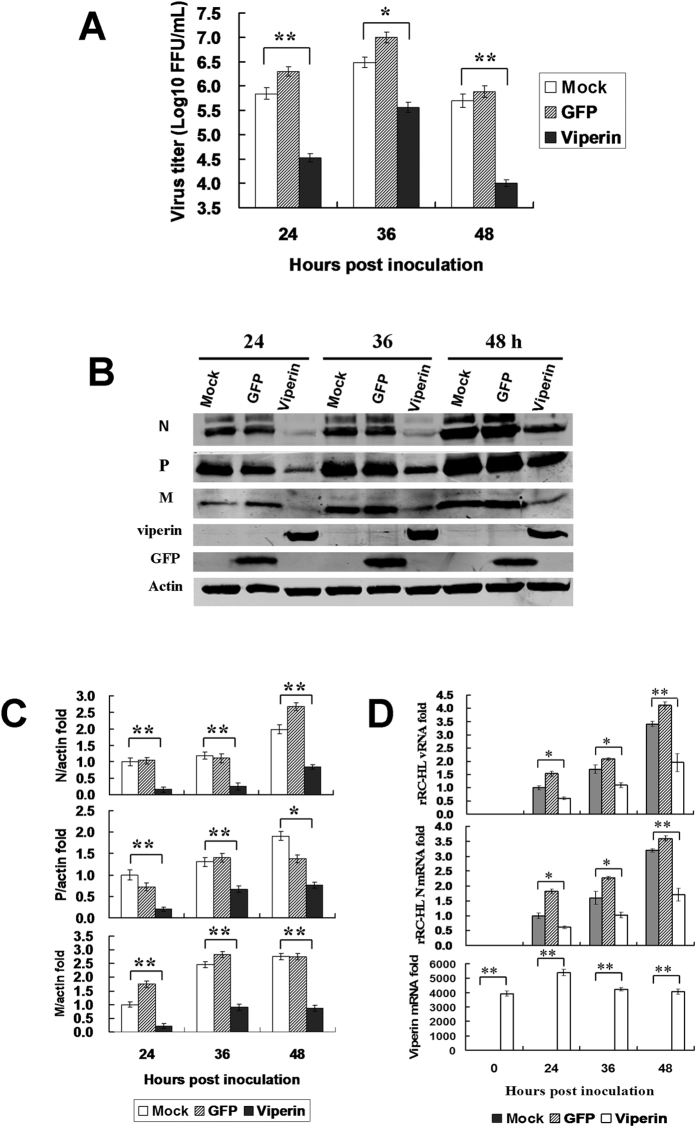
Viperin expression inhibits RABV replication. (**A**) Viperin inhibits RABV replication in viperin-eGFP-transfected BHK-21 cells. The viperin stably expressing BHK-21 cells were infected with rRC-HL at an MOI of 0.1. Virus titres were determined at 24, 36, and 48 hpi. (**B**) RABV proteins in the infected viperin stably expressing BHK-21 cells were detected by Western blotting. (**C**) The N protein/actin, P protein/actin and M protein/actin ratios in Figure 2F were measured using Li-Cor Odyssey 3.0 analytical software version 29. (**D**) RNA expression levels of viperin. rRC-HL vRNA and N mRNA expression levels were detected by qRT-PCR at 24, 36, and 48 hpi. Viperin-expressing BHK-21 cells were infected with rRC-HL at an MOI of 0.01. Data were normalized to β-actin expression and are presented as relative fold expression values to each control cell population infected with rRC-HL.

In another experiment, we generated a stably viperin-expressing BHK-21 cell line by transfecting the viperin-expressing plasmid peGFP-viperin. The viperin-expressing BHK-21 cells presented a bright green-fluorescence of enhanced green fluorescent protein (eGFP) fused with viperin that appeared to be localized within the cytoplasm (data not shown). The stably viperin-expressing BHK-21 cells were infected with rRC-HL at a multiplicity of infection (MOI) of 0.01, and the cell supernatant was used to titrate the virus titre. Viral replication was significantly inhibited following viperin expression. The titres of rRC-HL in GFP-viperin-expressing BHK-21 cells were 3.31 × 10^4^ FFU/mL, 3.36 × 10^5^ FFU/mL and 1.00 × 10^4^ FFU/mL at 24, 36 and 48 hpi; in contrast, the titres of rRC-HL in GFP-expressing BHK-21 cells reached 2.00 × 10^6^ FFU/mL, 1.00 × 10^7^ FFU/mL and 7.59 × 10^5^ FFU/mL (Fig. 2A). rRC-HL N, P and M protein expression levels were significantly reduced as a result of increased viperin expression (Fig. 2B,C). Meanwhile, RABV genomic RNA (vRNA) and N gene mRNA levels were also significantly reduced in the stably viperin-expressing cells (Fig. 2D). In addition, viperin also inhibited the replication of street RABV GX01 (Fig. S1A–D) and GXN119 (Fig. S1E–G) strains, which are representative isolates of groups I and III, respectively, from an epidemic in Guangxi, China^17^,^18^.

Based on previous studies^19^,^20^,^21^, the N-terminal amphipathic α-helical domain and the radical S-adenosylmethionine (SAM) domain (C83A/C87A/C90A) of viperin were also identified to have critical roles in inhibiting RABV replication (Fig. S2). To confirm whether the viperin-mediated inhibition of RABV infection occurs through the interaction between viperin and RABV proteins, pcDNA-N and pViperin, pcDNA-P and pViperin, pcDNA-M and pViperin plasmids were co-expressed in HEK293 cells, followed by co-immunoprecipitation assays with anti-viperin and anti-N, P and M monoclonal antibodies (MAbs). Viperin did not interact with N, P and M, specifically (Fig. S3). These results suggested that the viperin-mediated inhibition of RABV replication did not occur directly through binding to RABV proteins.

To understand if inhibition of RABV is a function of viperin expression levels, viperin was expressed in BSR cells by transfecting with 0.5–2.0 μg of pViperin plasmid for 12 h and then infecting the cells with rRC-HL at an MOI of 0.001. Intracellular RABV N, P and M protein expression levels were analysed by Western blotting. The results showed a linear relationship between the increase in pViperin plasmid transfection and decreased expression levels observed for the RABV N, P, and M proteins, indicating that viperin inhibits RABV replication in a dose-dependent manner (Fig. S4A,B).

### Viperin reduces cholesterol and sphingomyelin on the cellular membrane

**Figure 3:**
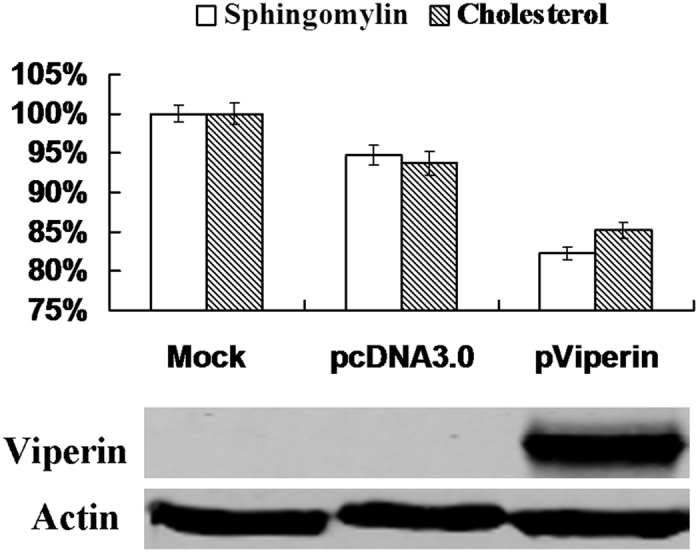
Viperin reduced cholesterol and sphingomyelin. The BSR cells were transfected with pViperin or the pcDNA3.0 plasmid for 24 h, and cholesterol and sphingomyelin contents in BSR cells were measured using kits. Simultaneously, viperin was detected by Western blotting. The data represent averages of three independent experiments. The total amounts of cholesterol and sphingomyelin were determined using colorimetric assays.

Cholesterol and sphingolipids are the main components of lipid rafts, the specific membrane microdomains required for viral entry, assembly, and budding of various viruses^22^,^23^. Therefore, we evaluated if the cholesterol and sphingolipids on the cellular membrane were impaired by viperin. The changes in cellular cholesterol and sphingomyelin were analysed by transfecting with pViperin. BSR cells were transfected with pViperin and pcDNA3.0, and the cholesterol and sphingomyelin levels were then assessed using a colorimetric assay. Both cholesterol and sphingomyelin levels were reduced to 85.19% and 82.24% in the BSR cells transfected with pViperin plasmid compared to normal cells (Fig. 3). However, the amounts of cholesterol and sphingomyelin in the BSR cells transfected with an empty vector pcDNA3.0 were reduced to 93.73% and 94.72%, respectively, compared to normal cells, suggesting that viperin reduced cholesterol and sphingomyelin levels on the cellular membranes of the BSR cells.

### Inhibitors of cholesterol and sphingomyelin affect RABV budding and release

**Figure 4:**
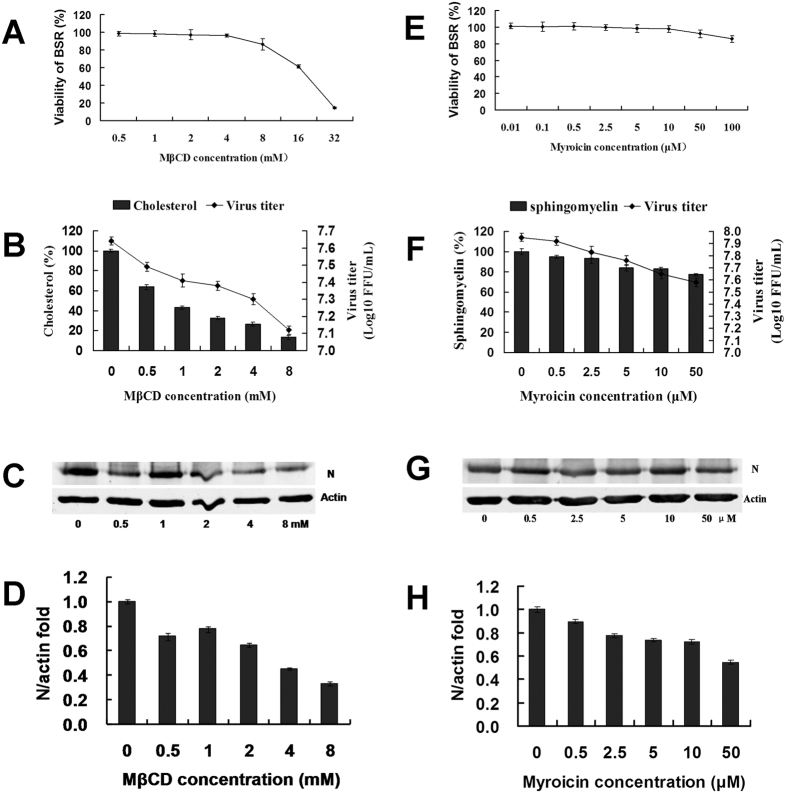
Inhibition of cholesterol and sphingomyelin affected RABV budding and release. (**A**) Effect of MβCD on BSR cell viability. BSR cells seeded in 96-well microplates were untreated or pre-treated with 0.5–8 mM MβCD for 1 h. The supernatants were removed and washed twice with PBS. An MTT assay was then performed. (**B**) Effect of MβCD on cholesterol and RABV replication. Cells seeded in six-well microplates were infected with rRC-HL at an MOI of 0.001 and then treated with 0.5–8 mM MβCD for 24 h at 37 °C. The supernatants were harvested for viral titration. The cells were assayed for cholesterol content using a cholesterol quantitation kit (40006; AAT Bioquest, Inc.) according to the manufacturer’s specfications. (**C**) Effect of MβCD on RABV replication. Based on A and B, a set of the cells was used to prepare cell lysates that were subjected to Western blot analysis for RABV N and β-actin protein expression. (**D**) The N protein/actin ratios in Fig. 3C were measured using Li-Cor Odyssey 3.0 analytical software version 29. The error bars were calculated from at least 3 independent inhibition tests. (**E**) Effect of myriocin on BSR cell viability. BSR cells were seeded in 96-well microplates and either untreated or pre-treated with 0.01–100 μm myriocin for 1 h. The supernatants were removed and washed twice with PBS. An MTT assay was then performed. (**F**) Effects of myriocin on sphingomyelin content and RABV replication. Cells seeded in six-well microplates were infected with rRC-HL at an MOI of 0.001 and then treated with 0.5–50 μm myriocin for 24 h at 37 °C. The supernatants were harvested for viral titration. The cells were assayed for sphingomyelin content using a sphingomyelin colorimetric assay kit (10009928). (**G**) Effect of myriocin on RABV replication. Based on D and E, another set of cells was used to prepare lysates that were then subjected to Western blotting analysis for RABV N and β-actin protein expression. (**H**) The N protein/actin ratios in Fig. 3G were measured using Li-Cor Odyssey 3.0 analytical software version 29.

In this experiment, MβCD, a cholesterol inhibitor, and myriocin, a sphingomyelin biosynthesis inhibitor, were used to first understand the effects on cell growth. MβCD and myriocin were dissolved in Dulbecco’s modified Eagle’s medium (DMEM) supplemented with 2% foetal calf serum (FCS) at 0.5–32 mM and 0.01–100 μM, respectively. The BSR cells were incubated with MβCD and myriocin for 24 h, respectively, and the viability of the treated BSR cells was determined by 3-(4,5-dimethylthiazol-2-yl)-2,5-diphenyltetrazolium bromide (MTT) assay. Both MβCD and myriocin could reduce BSR cell viability in a dose-dependent manner. When the concentration of MβCD reached 8 mM, it could significantly reduce BSR cell viability to 86.57% (Fig. 4A), and when the concentration of myriocin reached 50 μM, it could significantly reduce BSR cell viability to 92.13% (Fig. 4E).

To investigate if MβCD and myriocin affected RABV replication, BSR cells were pre-treated with 0.5–8 mM MβCD and 0.5–50 μM myriocin, respectively. At 2 h post-treatment at 37 °C, the cells were collected to prepare the lysates for assessment of cholesterol and sphingomyelin, respectively. The contents of both cholesterol and sphingolipids decreased in a dose-dependent manner. Treatment with 1 mM MβCD decreased the cholesterol to 43.22% the level in the control cells (Fig. 4B), and treatment with 50 μM myriocin decreased sphingolipids to 77.02% the level in the control cells (Fig. 4F). Thus, MβCD and myriocin could efficiently impair cholesterol and sphingolipids in BSR cells.

To investigate if the inhibitors of cholesterol and sphingomyelin affected RABV budding and release, BSR cells were infected with rRC-HL at an MOI of 0.001 and were then treated with serial concentrations of MβCD or myriocin for 24 h. The infected BSR cell cultures were used to measure the contents of cholesterol and sphingomyelin and were simultaneously used to detect RABV N protein by Western blotting; their supernatants were used to titrate RABV titres. RABV titres decreased as MβCD and myriocin concentrations increased (Fig. 4B,F). Furthermore, RABV N protein expression in the infected BSR cells cultures decreased as MβCD and myriocin levels increased (Fig. 4C,D,G,H). These results revealed that inhibitors of cholesterol and sphingolipids affected RABV budding and release.

### RABV induces TLR4 signalling pathways to regulate viperin in RAW264.7 cells

**Figure 5:**
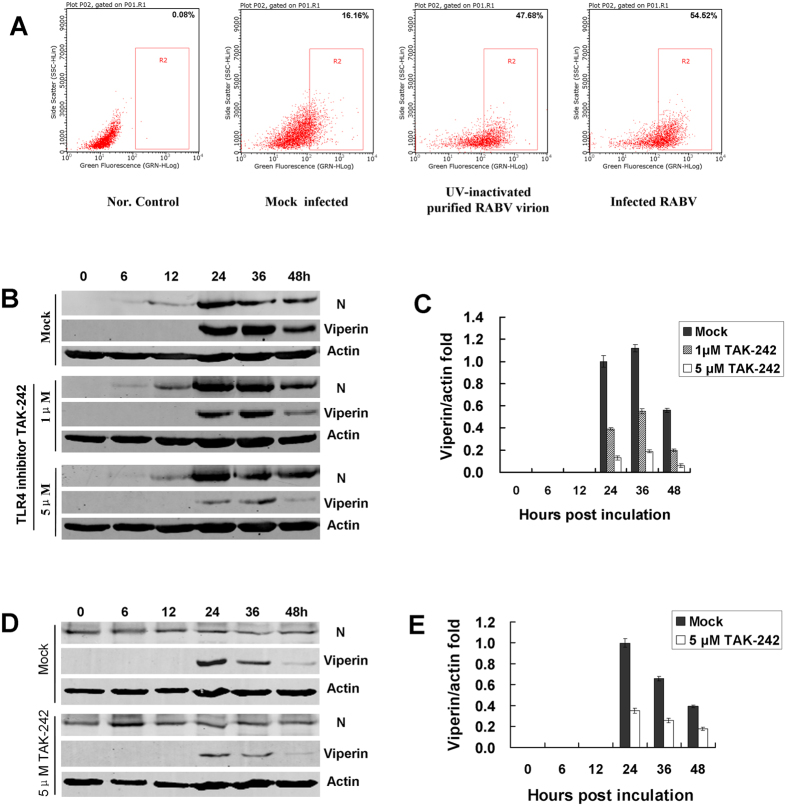
TLR4 is involved in RABV-inducted viperin regulation. (**A**) Altered TLR4 expression on membranes during RABV infection. RAW264.7 cells were mock-infected, UV-inactivated purified RABV or infected with rRC-HL at an MOI of 0.1 at 37 °C for 24 h, and cells were then analysed by flow cytometry. Data from triplicate experiments were then analysed using Guava soft 2.6 software. (**B**) RAW264.7 cells were infected with rRC-HL at an MOI of 0.1 and were then treated with 1.0 μM or 5.0 μM of TLR4-specific inhibitor TAK-242. The cell lysates were subjected to Western blotting analysis to detect viperin, RABV N protein, and β-actin protein expression. (**C**) The viperin protein/actin ratios in Figure 5D were measured using Li-Cor Odyssey 3.0 analytical software version 29. (**D**) The RAW264.7 cells were incubated with UV-inactivated purified RABV rRC-HL virion and were then treated with 5.0 μM of TLR4-specific inhibitor TAK-242. The cell lysates were subjected to Western blot analysis to detect viperin, RABV N protein, and β-actin protein expression. (**E**) The viperin/actin ratios in Fig. 5D were measured using Li-Cor Odyssey 3.0 analytical software version 29.

TLR4 was identified as recognizing lipopolysaccharides to activate interferon regulatory factor 3 (IRF3), resulting in IFNβ production^24^. In addition, TLR4 can also recognize various glycoproteins in viral envelopes, such as the fusion (F) protein of respiratory syncytial virus (RSV)^25^,^26^. Furthermore, the envelope proteins (EnV) of the retroviruses murine mammary tumour virus (MMTV) and Moloney murine leukaemia virus both interact with TLR4^27^. To determine whether RABV induced IFN-α/β expression through the TLR4 signal pathway, RAW246.7 cells were incubated with UV-inactivated purified RABV rRC-HL virion or were infected with RABV rRC-HL strain. TLR4 expression in the cells was subsequently analysed by flow cytometry at 24 hours post-incubation. The uninfected RAW264.7 cells incubated with fluorescein isothiocyanate (FITC)-labelled normal mouse IgG were used to normalisation (actual expression of 0.08%). The respective TLR4 expression levels in uninfected, UV-inactivated purified RABV rRC-HL virion and RABV-infected RAW246.7 cells were 16.16%, 47.68% and 54.52%. TLR4 expression in the RAW264.7 cells infected with RABV was 3.37-fold higher than that of the uninfected cells (Fig. 5A). In addition, TLR4 mRNA was up-regulated in RAW264.7 cells infected with RABV by 2.24-, 3.37- and 2.78-fold at 12, 24, and 36 hpi, respectively (data not shown), suggesting that TLR4 is involved in the process of the RABV infection-related induction of viperin expression in this cell line.

Furthermore, the TLR4-specific inhibitor TAK-242, a cell-permeable cyclohexenecarboxylate interacting with the adaptor molecules TIRAP and TRAM via directly binding to the intracellular Cys747 residue of TLR4^28^,^29^, was used to block the TLR4 signalling pathway. RAW264.7 cells were subsequently infected with rRC-HL at an MOI of 0.1. Viperin levels were obviously reduced by the TLR4-specific inhibitor TAK-242 (Fig. 5B,C). In addition, RAW246.7 cells inoculated with UV-inactivated purified RABV rRC-HL virion showed that RABV virion could be acted as a ligand binding to TLR4 and induced viperin. Furthermore, viperin levels induced by UV-inactivated purified RABV rRC-HL virion were obviously reduced by the TLR4-specific inhibitor TAK-242 (Fig. 5D,E). These findings suggested that RABV induces TLR4 signalling pathways to regulate viperin expression in RAW264.7 cells.

### Identification of other cellular factors in the TLR4 signal transduction pathway involved in RABV-induced viperin regulation in RAW264.7 cells

**Figure 6:**
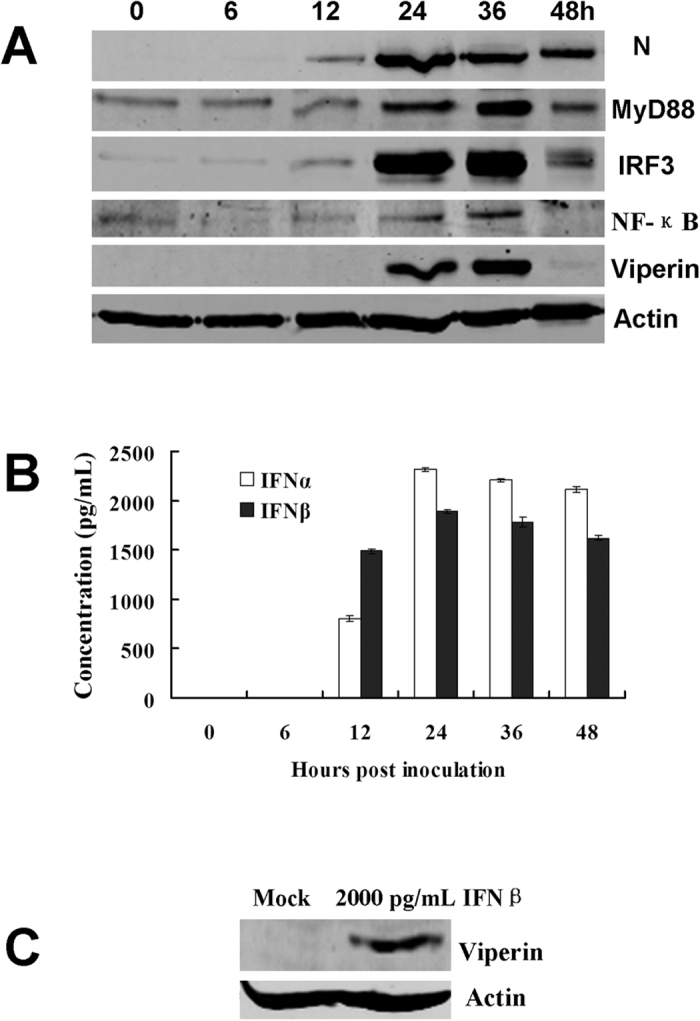
Identification of cellular factors in the TLR4 signal transduction pathway involved in RABV-inducted viperin regulation. (**A**) Detection of cellular factors in the TLR4 signal transduction pathway involved in RABV-induced viperin regulation. RAW264.7 cells were infected with rRC-HL at an MOI of 0.1 and were then used to prepare lysates. These lysates were subjected to Western blotting analysis to detect MyD88, IRF3, IFN-β, viperin, NF-κB (p65), RABV N and β-actin protein expression. (**B**) Based on (**A**), the supernatants were used to assess IFN-α/β contents using ELISA kits. (**C**) The RAW264.7 cells were treated with 2,000 pg/mL IFNβ for 24 h, and the cell lysates were then subjected to Western blotting analysis to detect viperin protein expression.

The above data confirmed that RABV induced IFN-α/β and viperin through TLR4. At the same time, the signalling factors, such as MyD88, IRF3 and nuclear factor κB (NF-κB), of the TLR4 transduction pathway were also monitored during RABV infection. Of these factors, MyD88 and IRF3 were up-regulated following RABV infection, which was consistent with RABV-induced IFN-α/β and viperin up-regulation (Fig. 6A–C).

**Figure 7:**
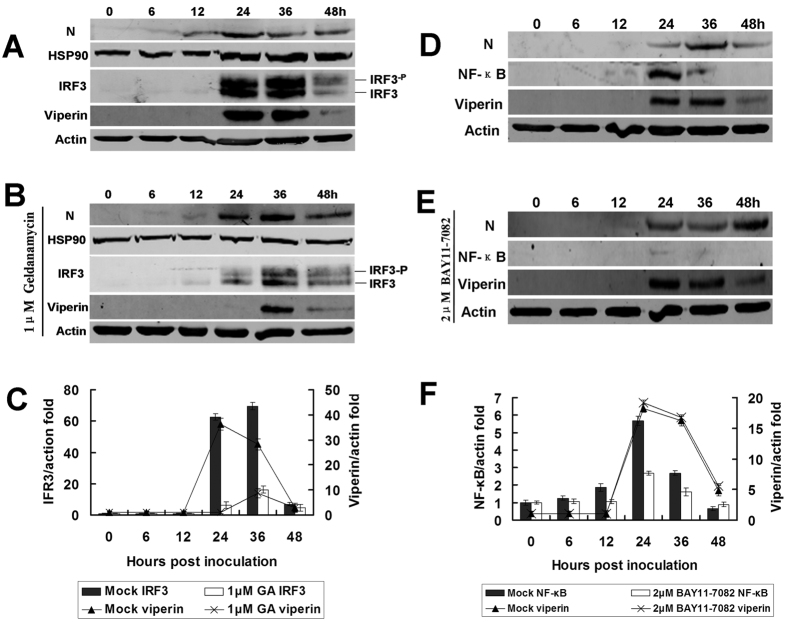
IRF3/HSP90 rather than NF-κB participates in viperin regulation. (**A**) *In vitro* infection with RABV affecting IRF3 expression. RAW264.7 cells were infected with rRC-HL at an MOI of 0.1, and DMSO (for dissolving geldanamycin [GA], an inhibitor of IRF3) was added as a mock control. The cell lysates were prepared and subjected to Western blotting analysis to detect IRF3, Hsp90, viperin, RABV N, and β-actin protein expression. (**B**) GA inhibits IRF3 and viperin expression. RAW264.7 cells were infected with rRC-HL at an MOI of 0.1 and were treated with 1.0 μM GA. The cell lysates were prepared and subjected to Western blotting analysis to detect IRF3, Hsp90, viperin, RABV N, and β-actin protein expression. (**C**) The IRF3 and viperin protein/actin ratios in Fig. 3A,B were measured using Li-Cor Odyssey 3.0 analytical software version 29. (**D**) Mock-treated RAW264.7 cells were infected with rRC-HL at an MOI of 0.1. The cell cultures were used to prepare lysates and were subjected to Western blotting analysis to detect NF-κB (p65), viperin, RABV N, and β-actin protein expression. (**E**) RAW264.7 cells were infected with rRC-HL at an MOI of 0.1 and then treated with the NF-κB (p65)-specific inhibitor BAY11-7082 to a final concentration of 2.0 μM. The cell cultures were used to prepare lysates and were subjected to Western blotting analysis to detect NF-κB (p65), viperin, RABV N, and β-actin protein expression. (**F**) The NF-κB and viperin protein/actin ratios in Fig. 3D, E were measured using Li-Cor Odyssey 3.0 analytical software version 29.

Because geldanamycin (GA) selectively inhibits heat-shock protein 90 (Hsp90), the chaperone of IRF3 phosphorylation^30^, as expected, Hsp90 and IRF3 expression and IRF3 phosphorylation were reduced following GA treatment (Fig. 7A–C). However, NF-κB levels changed only slightly upon RABV infection (Figs 6A and 7D). Another experiment using an NF-κB-specific inhibitor, BAY11-7082, demonstrated that NF-κB was not involved in viperin regulation (Fig. 7E,F).

## Discussion

Viperin inhibits the replication of different viruses via different mechanisms^14^. In this study, we found that RABV replication was inhibited in macrophage RAW264.7 cells. Further studies indicated that RABV was inhibited by viperin, an IFN-inducible protein (Figs 2 and 3). Therefore, viperin was identified as the first immune response factor to inhibit RABV, although it was previously reported to inhibit the budding and release of influenza A virus^13^, human immunodeficiency virus (HIV)^31^ and hepatitis C virus (HCV)^12^. We demonstrated that natural viperin in RAW264.7 cells inhibited rRC-HL and that the viperin expressed in transiently transfected BSR cells or in stably transfected BHK-21 cells also inhibited rRC-HL.

Further experimental results showed that cholesterol and sphingomyelin levels were reduced in transfected pViperin BSR cells (Fig. 3). This hypothesis was also supported by the use of inhibitors of cholesterol and sphingomyelin. MβCD, an inhibitor of cholesterol, and myriocin, an inhibitor of sphingomyelin, could reduce the respective cholesterol and sphingomyelin levels and also inhibited RABV replication (Fig. 4B,F). However, inhibition by MβCD and myriocin was not effective in pre-treated cells (Fig. S5), indicating that MβCD and myriocin did not efficiently affect RABV adsorption. Furthermore, RAW246.7 cells were inoculated with UV-inactivated purified RABV rRC-HL virion and demonstrated that RABV virion acted as a ligand of TLR4 to induce viperin (Fig. 5).

To understand how RABV induces viperin production in RAW264.7 cells, innate immune response receptor expression on RAW264.7 cells during RABV infection was assessed. Using a TLR4 inhibitor (TAK-242), TLR4 was identified to be involved in viperin regulation during RABV infection. Previous investigations found that IRF3 was an important transcriptional regulator of the antiviral immune response, mediating the expression of type I IFN and ISGs^32^,^33^. Thus, IRF3 was determined to be induced in RAW264.7 cells during RABV infection. Furthermore, the immune response factors MyD88, IRF3, and Hsp90 were identified as important components of the TLR4 signal transduction pathway and mediated the antiviral action of viperin (Figs 6 and 7A,B). Although NF-κB expression increased only slightly during RABV infection (Figs 6A and 7D), viperin expression was essentially not affected when the NF-κB inhibitor BAY11-7082 was administered (Fig. 7D–F), suggesting that NF-κB is not involved in viperin regulation.

**Figure 8:**
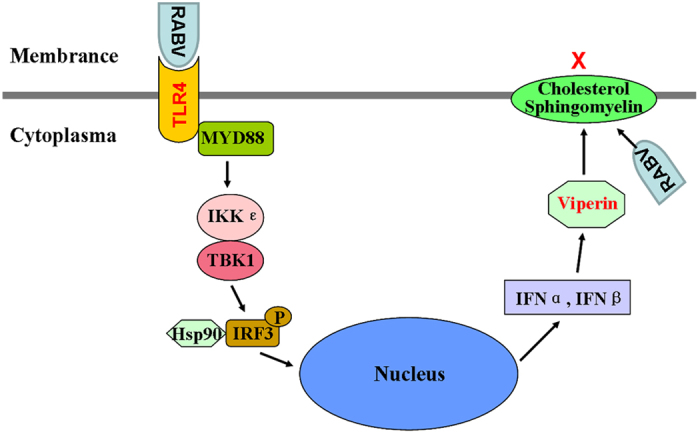
Proposed model of viperin-mediated inhibition of RABV replication and its up-stream signal regulation. The novel data from this study are hypothesized in this figure.

In this study, a multi-functional protein; i.e., viperin, was identified to inhibit RABV replication. To date, viperin is the first protein to be reported to affect RABV replication, even though it has been previously shown to affect influenza A virus, HIV and HCV. The function of viperin-mediated RABV inhibition in RAW264.7 cells was determined to operate via cholesterol and sphingomyelin reducing on the cellular membrane. Furthermore, TLR4 was verified as an upstream regulator in the signal transduction pathway. The novel data from this study are hypothesized in Fig. 8. These findings will be helpful for the development of new antiviral strategies using viperin in the future.

## Materials and Methods

### Ethics statement

All animal experiments performed in this paper were conducted according to the National Guideline on the Humane Treatment of Laboratory Animals Welfare (MOST of People’s Republic of China, 2006) and approved by the Animal Welfare and the Animal Experimental Ethical Committee of Guangxi University (No. Xida-kezi2000138). All animal experimental methods were carried out in accordance with the approved guidelines. The husbandry procedures were conducted in compliance with the Animal Welfare Act and the Guide for the Care and Use of Laboratory Animals. All experimental protocols including samples collection protocol were approved by the Department of Veterinary Administration of Guangxi Province. The brain samples of normal dogs were collected and provided by the Guangxi Centre for Animal Disease Control and Prevention (the Animal CDC). Mice used for viral isolation by the mouse inoculation test (MIT) were observed for 28 days post-injection, and then were euthanized in a container by halothane inhalant.

### Cells and virus

The neuroblastoma cells (NA) were purchased from the Chinese Academy of Sciences Shanghai Institutes for Biological Sciences-Cell Resource Centre. BSR cells, a clone of BHK cells, were kindly provided by Dr. Zejun Li (Shanghai Veterinary Research Institute, Chinese Academy of Agricultural Sciences, China). Baby hamster kidney cells (BHK-21) and mouse macrophage cells (RAW246.7) were stored in the Lab of Animal Infectious Diseases, College of Animal Science and Veterinary Medicine Science, Guangxi University. All cells were grown in DMEM (GIBCO, USA) containing 10% FCS.

BHK^-GFP^ or BHK^-viperin-GFP^ cells were constructed by transfecting peGFP-N1 or peGFP-viperin plasmids into BHK-21 cells, respectively, and were screened by G418 at least three times. These stably GFP- or viperin-GFP-expressing BHK cells were maintained in DMEM supplemented with 10% FCS and 500 μg/mL of G418.

The rabies virus rRC-HL was rescued from an infectious cDNA clone pRC-HL (kindly provided by Professor Minamoto, Gifu University, Japan) based on the fixed RABV RC-HL strain used to vaccinate animals in Japan^34^. The street rabies virus isolates GX01 and GXN119 were obtained from the brains of dogs in Guangxi, China and inoculated into 4-week-old mice; the strains were then prepared from mouse brains in our laboratory^17^,^18^.

RABV virions were purified by referencing the Sokol’s method^35^. Briefly, monolayer cultures of BSR cells were prepared and infected with RABV rRC-HL strain at a multiplicity of infection (MOI) of 0.01. The cells supernatant was harvested at 48h post-inoculation, and then the cells supernatant was centrifuged at 8000 rpm for 30 min at 4 °C, the sediment was removed. The RABV virions were precipitated by adding zinc acetate to a final concentration of 0.02 M to the cells supernatant at 4 °C for 1 h, and harvested by centrifugation at 8000 rpm for 1 h, and the pellet was suspended in a saturated solution of ethylenediaminetetraacetate (EDTA)-Tris, pH8.0. The suspension containing RABV virions was centrifuged at 1000 rpm for 5 min and the sediment was removed. The suspension was centrifuged to precipitate RABV virions in 30000 rpm for 3 h at 4 °C, and the pellet was resuspended with NTE buffer (0.13 M NaCl, 0.05 M Tris, 0.001 M EDTA). And then the resuspension containing RABV virions was layered onto 10–60% (w/v) sucrose density gradient in NTE buffer for centrifugation at 30000 rpm for 90 min. The RABV virion band was collected and dissolved in NTE buffer, and recovered by centrifugation at 30000 rpm for 3 h at 4 °C. Finally, the purified RABV virions were suspended in NTE buffer.

### Construction of viperin- or mutant-expressing plasmids

To generate a plasmid for expressing viperin, total RNA was extracted from mouse brains and was used to amplify the viperin gene by RT-PCR with specific primers (Table S1). The PCR product was cloned into the pMD-18T plasmid to construct recombinant pMD-viperin. The ORF of the viperin gene was subsequently subcloned into the expression plasmids pcDNA3.0/MCS and peGFP-N1 to construct pViperin and peGFP-viperin, respectively. Two other mutants containing the 43–361 amino acid region, the domain that mediates targeting of viperin to the cytosolic side of the endoplasmic reticulum (ER) and lipid droplets^20^, and the SAM domain, which is homologous to the MoaA motif present in the family of radical SAM enzymes, which uses SAM as a cofactor to bind to proteins containing iron-sulphur clusters via the CxxxCxxC motif^21^, were also constructed. In addition, the three expression plasmids, pcDNA-N, pcDNA-P, and pcDNA-M, which express the nucleoprotein (N), phosphoprotein (P) and matrix (M) proteins of the RABV rRC-HL strain, were constructed.

### Total RNA extraction and quantitative RT-PCR (qRT-PCR)

Total RNA was extracted using RNAeasy Mini Kits (Qiagen, Cat. No. 74104) according to the manufacturer’s instructions. On-column DNase digestion was performed using the RNase-Free DNase Set (Qiagen, Cat. No. 79254). The integrity of total RNA was analysed using a Nanodrop 1000 spectrophotometer (Thermo, USA).

qRT-PCR was performed using the SYBR Green method, as previously described^36^. Briefly, 1 μg of total RNA served as the template for the first-strand cDNA synthesis in a reaction using an oligo(dT) primer and Moloney murine leukaemia virus (MMLV) reverse transcriptase under the conditions described by the manufacturer. A LightCycler 480 PCR detection system (Roche Diagnostics Ltd.) was used for the quantitative assessment of RABV N and viperin mRNA under standard cycling conditions. β-actin gene expression was assessed as a control for all reactions. The primer sequences are listed in Table S1.

### ELISA for IFN-α/β

An enzyme-linked immunosorbent assay (ELISA) was performed using a mouse IFN-α ELISA Kit (eBioscience, San Diego, CA., USA) and a mouse IFN-β ELISA Kit (PBL InterferonSource, Piscataway, NJ, USA) following the manufacturer’s instructions. The RAW246.7 cells were infected with rRC-HL at an MOI of 0.1. After 0, 6, 12, 24, 36 and 48 hpi, the supernatants of the infected RAW246.7 cells were collected, and the IFN-α/β concentrations in the supernatants were measured with an ELISA kit.

### Western blotting

Western blots were performed as previously described^36^. RAW264.7 cells were washed twice with PBS and lysed in lysis buffer (10 mM Tris pH 7.4, 100 mM NaCl, 1.5 mM MgCl_2_, 0.5% NP-40). The lysate was cleared by centrifugation at 8,000 rpm for 5 min at 4 °C. The lysate was subjected to 10% sodium dodecyl sulphate polyacrylamide gel electrophoresis (SDS/PAGE), and the proteins were then transferred to polyvinylidene difluoride (PVDF) membranes (Millipore, Billerica, MA) that were blocked for 60 min at room temperature in 1 × TBST buffer (50 mM Tris-HCl pH7.4, 250 mM NaCl, 0.1% Tween-20) containing 1% non-fat milk powder. The membranes were incubated overnight at 4 °C with primary antibodies. An anti-viperin monoclonal antibody (Mab; ab107359), along with anti-Hsp90 MAb (ab13492), anti-MyD88 polyclonal Ab (ab135693), anti-IRF3 (ab68481), anti-NF-κB (p65; ab90532) antibodies, were purchased from Abcam Ltd (USA). Anti-rabies virus matrix protein (M) MAb (53-D-3-3) and anti-rabies virus nucleoprotein (N) MAb (N13-27) antibodies were kindly provided by Dr. Minamoto (Gifu University, Japan). Anti-GFP polyclonal Ab (50430-2-AP) was purchased from Proteintech Group (China). Anti-β-actin MAb (cw0096A) was purchased from Beijing ComWin Biotech (China). An anti-rabies phosphoprotein (P) polyclonal Ab was prepared in our laboratory. After 5 washes with 1 × TBST, the membranes were incubated with the corresponding secondary antibody for 1 h at room temperature, followed by visualization of the immunoreactive bands using a 5-bromo-4-chloro-3-indolyl-phosphate/nitro blue tetrazolium (BCIP/NBT) kit (Beyotime, China). Quantification of immunoreactivity was performed by densitometric analysis using an Odyssey scanner (Li-Cor, Lincoln, NE, USA).

### MTT assay

An MTT assay was performed as follows: BSR cells were seeded in 96-well microplates with six replicates, either untreated or pre-treated with MβCD (Sigma, USA) or myriocin (Sigma, USA), and incubated at 37 °C and 5% CO_2_. After incubation for 24 h post-treatment, the supernatants were discarded and washed three times with PBS, and the MTT reagent (5 mg/mL in PBS) was then added to each well and incubated at 37 °C for 3 h, after which the supernatant was removed. Subsequently, 200 μL dimethyl sulphoxide (DMSO) was added and incubated at 37 °C for 30 min. Finally, the plates were read on an Imark Microplate Reader (Bio-Rad, USA). The data were calculated as coverage values of three repeated experiments.

### Assessment of cholesterol and sphingomyelin content on the cellular membrane

To quantify cholesterol and sphingomyelin on the membranes of cells either infected or uninfected with RABV, treated or untreated with MβCD (Sigma, USA) and myriocin (Sigma, USA), BSR cells were seeded in six-well microplates and either untreated or pre-treated with MβCD or myriocin for 1 h at 37 °C. After three washes with PBS, 10^6^ cells were harvested and lysed and were then used to measure the content of cholesterol or sphingomyelin using a cholesterol quantification kit (40006; AAT Bioquest, USA) or a sphingomyelin colorimetric assay kit (10009928; Cayman Chemical, USA) according to the manufacturer’s specifications. Experiments were repeated in triplicate.

### Flow cytometric analysis

Monolayer cultures of 2 × 10^5^ RAW246.7 cells were mock-infected, UV-inactivated purified RABV virion incubated, and infected with RABV rRC-HL strain at an MOI of 0.1 at 37 °C for 1 h. After removed the inoculum and washed the cells with PBS for three times, the cells were incubated in DMEM containing 2% FCS at 37 °C for 24 h. Then, cells were incubated with 0.5% trypsase in PBS for 5 min at 37 °C and harvested. The cells were incubated with FITC-conjugated TLR4 mAb (ab45126) for 1 h at 4 °C in PBS containing 0.5% bovine serum albumin (BSA). Finally, the cells were analysed in a Guava Flow Cell System (Guava easyCyte HT, Millipore). The data were analysed using guavaSoft 2.6 software. Samples were run in triplicate.

### Statistical analysis

Statistical significance was determined with a one-way analysis of variance (ANOVA), with P values of <0.05 considered statistically significant. Results are presented as the mean ± standard deviation (SD). Results were obtained from at least three independent experiments.

## Additional Information

**How to cite this article**: Tang, H.-B. *et al.* Viperin inhibits rabies virus replication via reduced cholesterol and sphingomyelin and is regulated upstream by TLR4. *Sci. Rep.* **6**, 30529; doi: 10.1038/srep30529 (2016).

## Supplementary Material

Supplementary Information
