# Supplementary material for: Viperin inhibits rabies virus replication via reduced cholesterol and sphingomyelin and is regulated upstream by TLR4
Source: Sci Rep. 2016 Jul 26;6:30529. doi: 10.1038/srep30529 (PMC4960569; doi:10.1038/srep30529)
Supplement: Supplementary Information [file srep30529-s1.doc]

Supplementary Information

Viperin inhibits rabies virus replication via reduced cholesterol and sphingomyelin and is regulated upstream by TLR4

Hai-Bo Tang^1,2^, Zhuan-Ling Lu^1,2^, Xian-Kai Wei^1,2^, Tao-Zhen Zhong^2^, Yi-Zhi Zhong^2^, Ling-Xuan Ouyang^2^, Yang Luo^2^, Xing-Wei Xing^2^, Fang Liao^2^, Ke-Ke Peng^2^, Chao-Qian Deng^2^, Nobuyuki Minamoto^2^, Ting Rong Luo﹡^1,2^

1. State Key Laboratory for Conservation and Utilization of Subtropical Agro-bioresources, Guangxi University, Nanning 530004, Guangxi, China

2. Laboratory of Animal Infectious Diseases, College of Animal Sciences and Veterinary Medicine, Guangxi University, Nanning 530004, Guangxi, China

Supplementary Figure 1

**
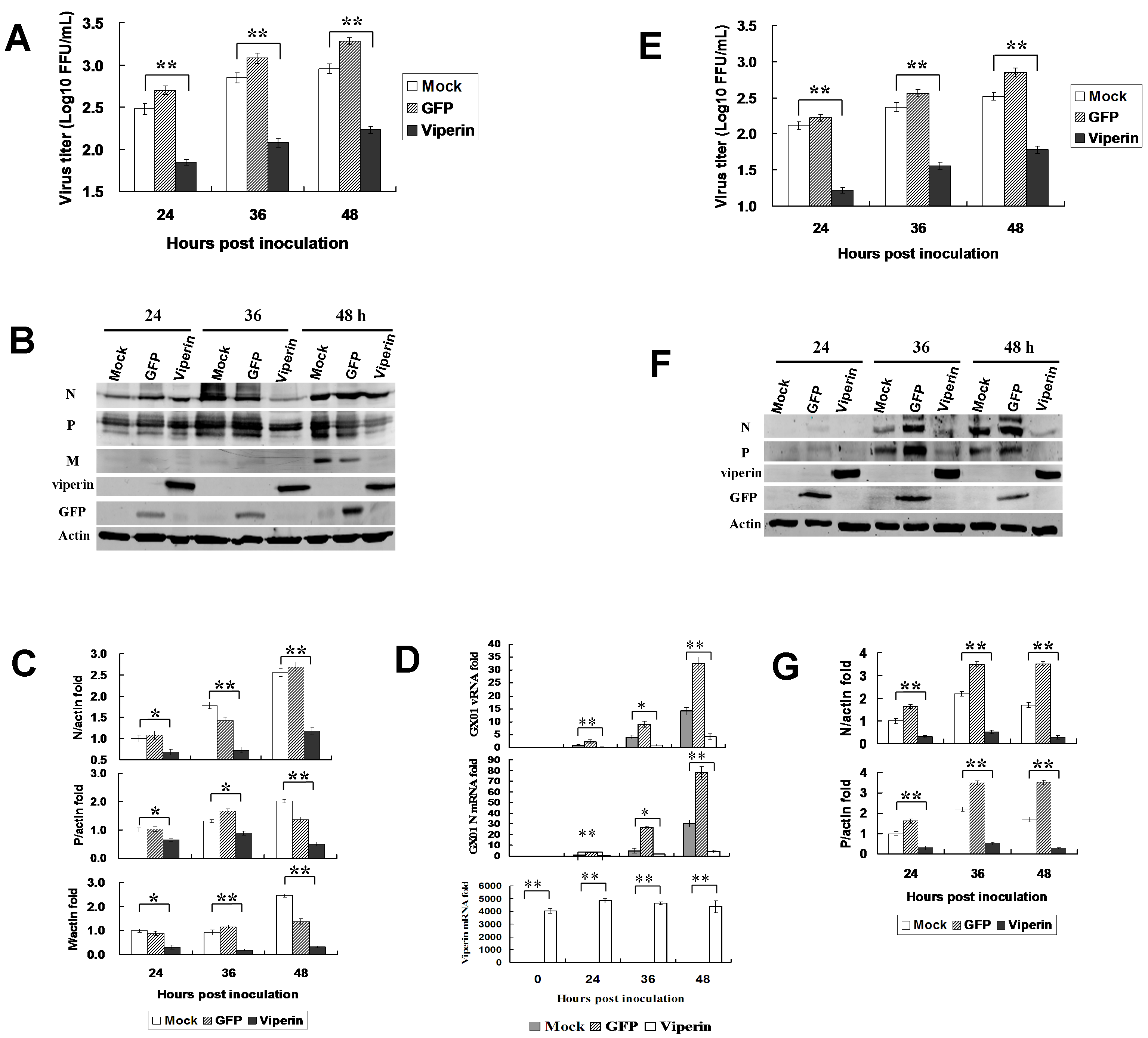
**

Supplementary Figure 1. Viperin inhibits replication of virulent street RABV.

(A) Viperin inhibits street RABV GX01 replication. The mock or stably viperin-expressing BHK-21 cells were infected with 0.01 MOI of the street RABV GX01 isolate which were isolated from Guangxi, China. The supernatant was used to detect virus titres at 24, 36, and 48 hpi by IFA.

(B) Viperin inhibits the synthesis of the RABV GX01 protein. The mock or stably viperin-expressing BHK-21 cells were infected with 0.01 MOI of street RABV GX01. The infected cell cultures were used to prepare lysates and subjected to detect viral protein by Western blotting.

(C) The N protein/actin, P protein/actin and M protein/actin ratios in Figure 2B were measured by using Li-Cor Odyssey 3.0 analytical software version 29.

(D) Viperin inhibits the synthesis of the RABV GX01 vRNA. The mock or stably viperin-expressing BHK-21 cells were infected with 0.01 MOI of street RABV GX01. The infected cell cultures were used to extract total RNA. The vRNA and N mRNA of GX01 were measured at 24, 36, and 48 hpi by qRT-PCR.

(E) Viperin inhibits street RABV GXN119 replication. The mock or stably viperin-expressing BHK-21 cells were infected with 0.01 MOI of street RABV GXN119 isolate which were isolated from Guangxi, China. The supernatant was used to detect virus titres at 24, 36, and 48 hpi by IFA.

(F) Viperin inhibits the synthesis of the RABV GXN119 protein. The mock or stably viperin-expressing BHK-21 cells were infected with 0.01 MOI of the street RABV GXN119. The infected cell cultures were used to prepare lysates and subjected to detect viral protein by Western blotting.

(G) The N protein/actin and P protein/actin ratio in Figure 2F were measured by Li-Cor Odyssey 3.0 analytical software version 29.

Supplementary Figure 2

**
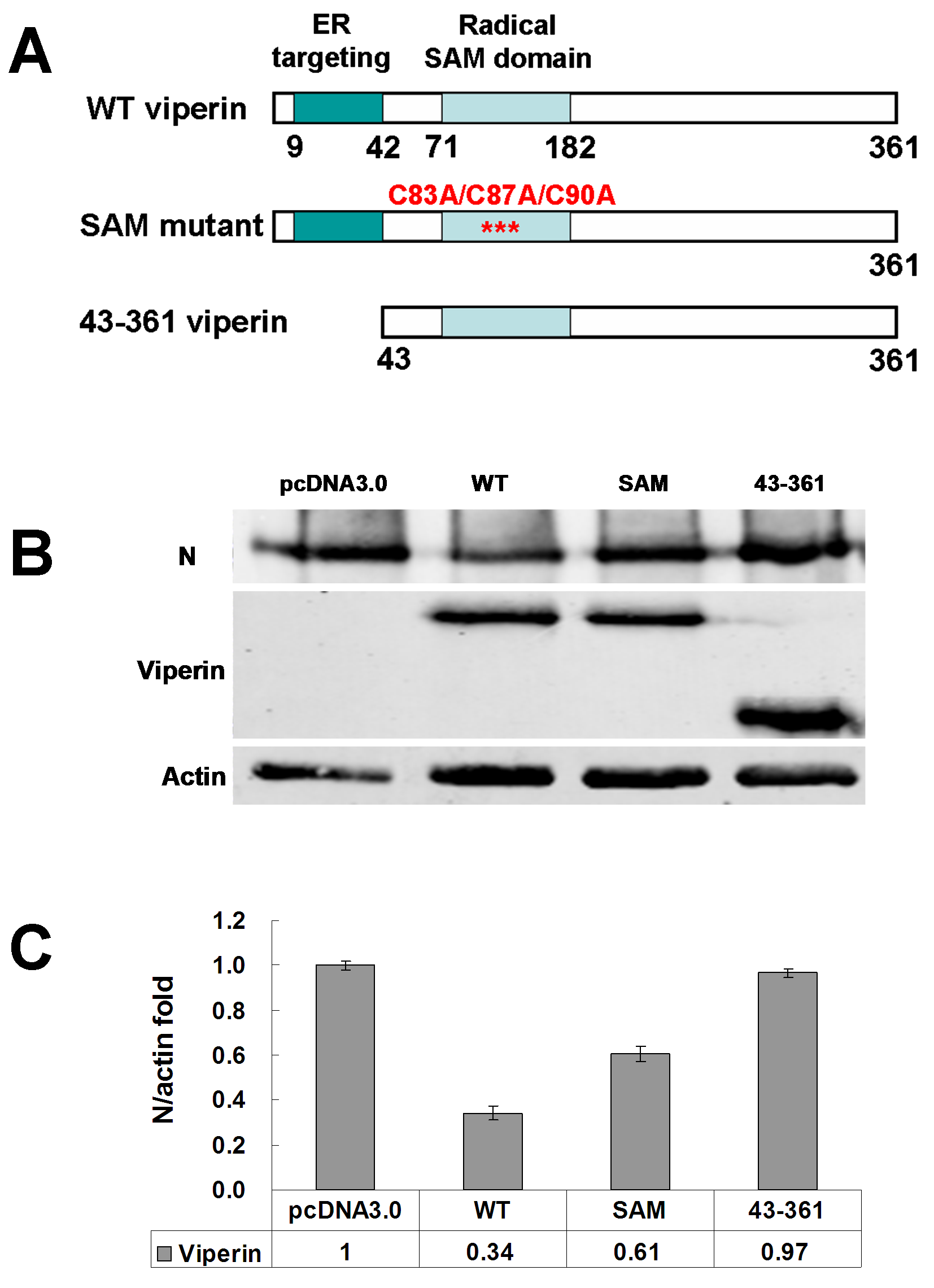
**

Supplementary Figure 2. Identification of the functional domain of viperin

(A) Schematic representation of the organization of viperin and construction of mutants. Viperin contains an ER targeting N-terminal amphipathic α-helical domain (1–42 amino acids, aa) and a radical SAM catalytic domain (77–209 aa). The fragments of wildtype (WT), SAM domain mutant (C83A/C87A/C90A), and a truncated mutant (43–361 aa) of viperin gene are constructed and cloned into the pcDNA3.0.

(B) The plasmids pViperin, pcSAM, and pcVip43-361 were transfected into BSR cells for 12h and then infected with 0.001 MOI of rRC-HL. Cells were harvested to prepare lysates at 24 hpi and subjected to analyze RABV N protein by Western blotting.

(C) The N protein/actin ratio in Figure S2B was measured by Li-Cor Odyssey 3.0 analytical software version 29.

Supplementary Figure 3


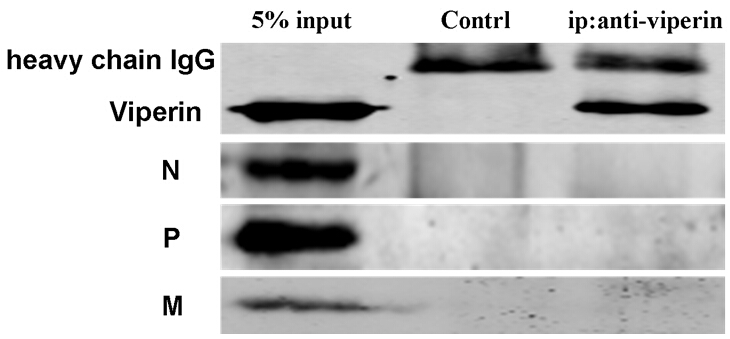


Supplementary Figure 3. Viperin does not bind to RABV N, P and M proteins

The couple pcDNA-N and pViperin, pcDNA-P and pViperin, pcDNA-M and pViperin plasmids were co-transfected in HEK293 cells, respectively, followed by co-immunoprecipitation assay with anti-viperin and anti-N, P and M MAbs.

Supplementary Figure 4

**
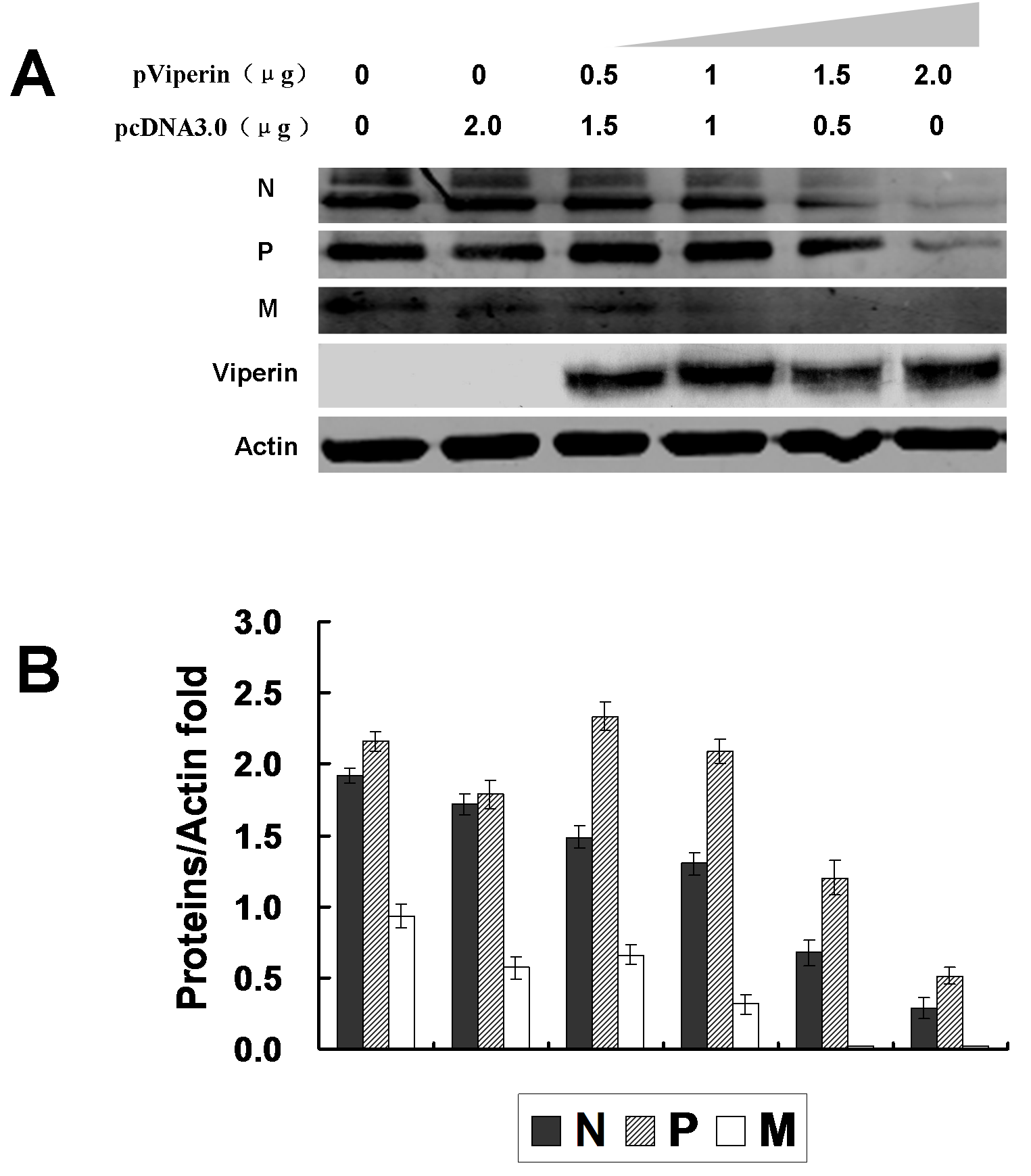
**

Supplementary Figure 4. Viperin inhibits RABV replication in a dose-dependent manner.

(A) The BSR cells were transfected with increasing amounts of pViperin plasmid for 12 hours and then infected with 0.001 MOI of rRC-HL. The cell cultures were harvested to prepare lysates at 24 hpi and the RABV N, P and M proteins were analyzed by Western blotting.

(B) The N, P and M proteins/actin ratio in Figure S4A were measured by Li-Cor Odyssey 3.0 analytical software version 29.

Supplementary Figure 5

**
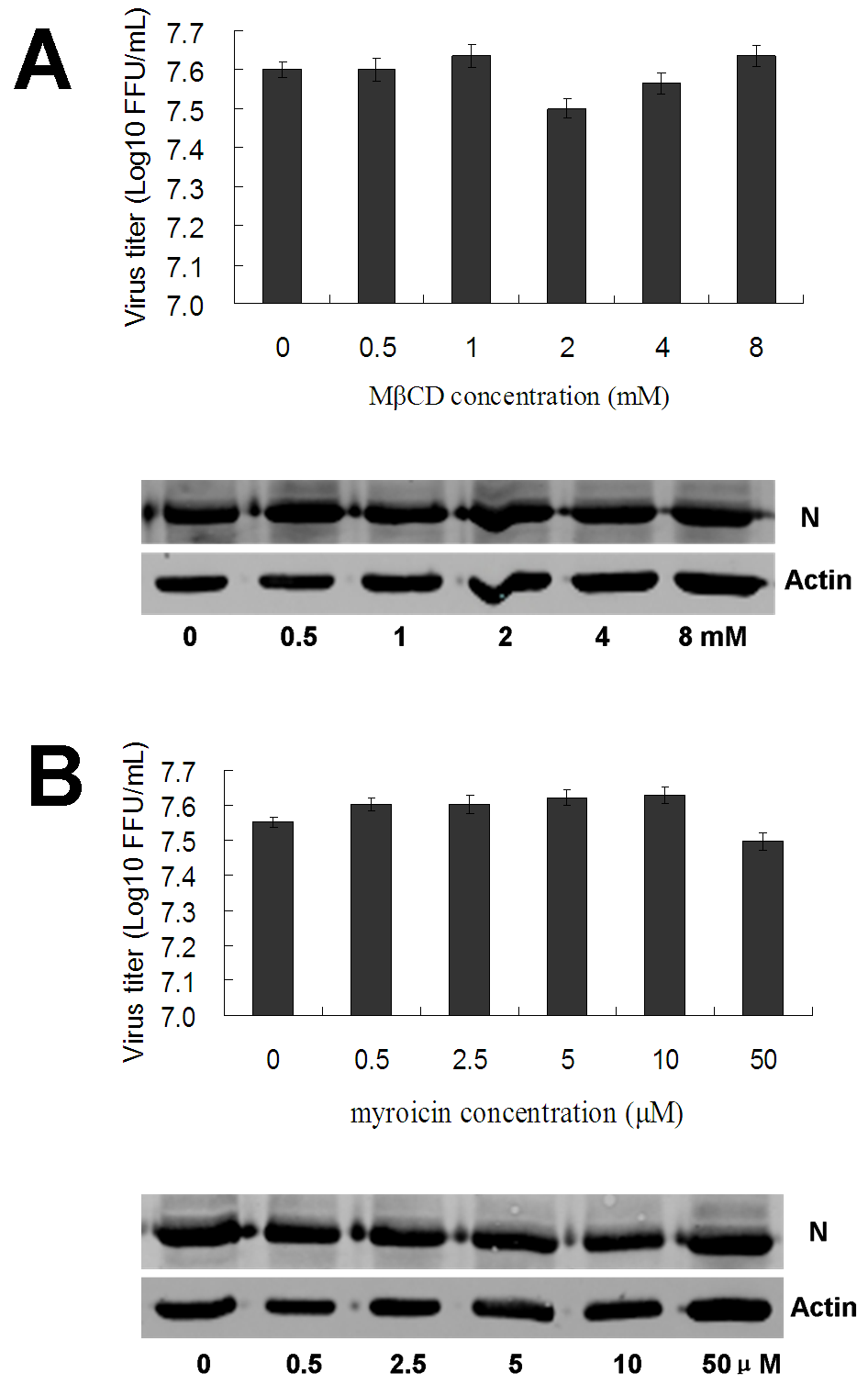
**

Supplementary Figure 5. Effect of pre-treatment with cholesterol and sphingolipid inhibitors on RABV adsorption.

(A) Effect of pretreated with MβCD, inhibitor of cholesterol, RABV adsorption. The BSR cells were pretreated with 0.5-8 mM MβCD for 1h, then the MβCD was removed and washed with DMEM for three times. The BSR cells were infected with 0.001 MOI of rRC-HL for 24h. The supernatants were used to viral titration in BHK-21 cells, the cell cultures were used to prepare lysates and subjected to detect RABV N protein and β-actin by Western blotting.

(B) Effect of pretreated with myriocin, inhibitor of sphingolipid, RABV adsorption. The BSR cells were pretreated with 0.5-50 μM myriocin for 1h, then the myriocin was removed and washed with DMEM for three times. The BSR cells were infected with 0.001 MOI of rRC-HL for 24h. The supernatants were used to viral titration in BHK-21 cells, the cell cultures were used to prepare lysates and subjected to detect RABV N protein and β-actin by Western blotting.

**Supplementary Table 1. Primers used in this experiment.**

| **Gene** | **Primers** | **Sequences** | **Position**  **(nt)** | **Aim** | **Length (bp)** |
| --- | --- | --- | --- | --- | --- |
| Viperin | m-vip U | 5'-GCCTATCACCATGGGGATGC-3' | 24-43 | RT-PCR | 1155 |
|  | m-vip L | 5'-GATAGCTGGGCGTGAATGT-3' | 1160-1178 |  |  |
| peGFP-viperin | Vip-gfp1 | 5'-AGAT***CTCGAG***GCCTATCACCATG-3' (**XhoI**) | -10-2 | subcloned | 1097 |
|  | Vip-gfp2 | 5'-AGCC***CTGCAG***CCAGTCCAGCTTCAG-3' (**Pst I**) | 1072-1086 |  |  |
| pviperin | 3.0 vip1 | 5'-CACC***GGATCC***ATGGGGATGCTGG-3' (**BamH I**) | 1-13 | subcloned | 1089 |
|  | 3.0vip2 | 5'-AGCC***CTCGAG***TCATCACCAGTCCAGCTTC-3' (**Pst I**) | 1074-1089 |  |  |
| pcVip43-361 | vip43-361 U | 5'-***GGATCC***GCCACCATGCCCGGGAAGGAACAGC-3' (**BamH I**) | 130-145 | subcloned (mutation) | 960 |
|  | vip43-361 L | 5'-AGCC***CTCGAGT***CATCACCAGTCCAGCTTC-3' (**Pst I**) | 1074-1089 |  |  |
| pcSAM (C83A/C87A/C90A) | 3.0vip-83 | 5'-CAGGCCAACTACAAAGCTGGCTTCGCCTTC-3' | 247—276 | mutation |  |
|  | 3.0vip-87 | 5'-GAAGGCGAAGCCAGCTTTGTAGTTGGCCTG-3' | 247—276 |  |  |
| Q-Rabies N gene | QN1 | 5'-GGCATTGGCAGATGATGGAACT-3' | 1137—1158 | qRT-PCR | 163 |
|  | QN2 | 5'-GGCTTGATGATTGGAACTGACTGA-3' | 1276—1299 |  |  |
| Q-Viperin | Qvip1 | 5'-ATTAATCGCTTCAACGTGGAC-3' | 673—693 | qRT-PCR | 258 |
|  | Qvip2 | 5'-GCGCATATATTCATCTAGGA-3' | 911—930 |  |  |
| Q-β-actin | Qβ-actin1 | 5'-AAGACCTCTATGCCAACACAGT-3' | 872—893 | qRT-PCR | 219 |
|  | Qβ-actin2 | 5'-CATCGTACTCCTGCTTGCTGAT-3' | 1069—1090 |  |  |
